# Difference analysis in prevalence of incidental pancreatic cystic lesions between computed tomography and magnetic resonance imaging

**DOI:** 10.1186/s12880-019-0341-5

**Published:** 2019-05-24

**Authors:** Shuo Zhu, Wen-Tao Wang, Xiao-Sha Shang, Ting Ni, Wen-Chuan Wu, Wen-Hui Lou, Meng-Su Zeng, Sheng-Xiang Rao

**Affiliations:** 1 Department of Radiology, Zhongshan Hospital, Fudan University, and Shanghai Institute of Medical Imaging, 180 Fenglin Rd., Shanghai, 200032 China; 2 Department of General Surgery, Zhongshan Hospital, Fudan University, Shanghai, China

**Keywords:** Pancreatic cyst, Computed tomography, Magnetic resonance imaging, Prevalence

## Abstract

**Background:**

The purpose was to investigate the difference of detection rate of incidental pancreatic cystic lesions (PCLs) with computed tomography (CT) and magnetic resonance imaging (MRI) and to compare the difference between CT and MRI and to explore the effect of this difference on surgical resection.

**Methods:**

We reviewed the diagnostic reports for incidental PCLs between 2013 and 2016. Images of PCLs would be re-evaluated. Clinical and imaging data were recorded. The chi-square and independent t-test were conducted for categorical and continuous variables.

**Results:**

The prevalence of PCLs was 1.91% (1038/54210) and 3.36% (1282/38099) on CT and MRI respectively, and increased with increasing age (*P* < 0.001). No significant differences were found in the annual prevalence of PCLs on CT (*P* = 0.796) and MRI (*P* = 0.213) from 2013 to 2016 while the number of examinations was increasing every year. The annual detection rate of MRI for small PCLs (< 20 mm) was significantly higher than CT (*P* < 0.001), but was not significantly different for large PCLs (≥20 mm). The rate of surgical resection of PCLs (≥20 mm) in MRI group was higher than CT (55.2% vs. 37.0%, *P* < 0.001).

**Conclusions:**

The detection rate of PCLs on CT and MRI tended to be stable despite increasing scan volumes. Female had a slightly more frequency of PCLs than male. MRI detected more small PCLs(< 20 mm) and had higher impact on surgical resection of large PCL(≥20 mm) compared with CT.

## Background

With increasing examinations of computed tomography (CT) and magnetic resonance imaging (MRI), pancreatic cystic lesions (PCLs) are identified with more frequency, often as an incidental finding for a condition unrelated to the pancreas. The detection rate of PCLs in population was reported ranging from 2.4 to 49.1% of various types of modalities [1–7], which may be caused by the different use of modalities and selection bias for a relatively small population.

PCLs comprise a spectrum of pathological types, ranging from completely benign to frank malignancy. Some cysts have malignant potential transformation into mucin-producing adenocarcinoma, but the rate was very low (33.2 per 100,000) [8]. The previous report noted that the mortality rate of PCLs resection was 2.1% [9]. However, it is sometimes difficult to differentiate benign cysts from malignancy by CT or MRI. This situation makes patients who have benign lesions being exposed to the risk of resection. Moreover, there was not any consensus on management of PCLs [10–12]. In clinical practice, which cyst needs surgical invention or imaging follow-up should be determined.

The increasing number of PCLs would be caused by advanced imaging modalities or an increase in real incidence. Due to the advantage of soft-tissue contrast and magnetic resonance cholangiopancreatography (MRCP), MRI is supposed to have higher detection rate in comparison with CT. However, whether this difference influences clinical management is still unknown. Realizing the real prevalence of pancreatic cysts in the general population is essential to understand its natural history. The purpose of the study was to investigate the detection rate of incidental PCLs on CT and MRI and to compare the difference between CT and MRI and to explore the effect of this difference on surgical resection.

## Methods

This was a retrospectively study approved by the Ethics Committee at Zhongshan Hospital of Fudan University. Requirement for informed patient consent was waived.

### Subject

**Fig. 1:**
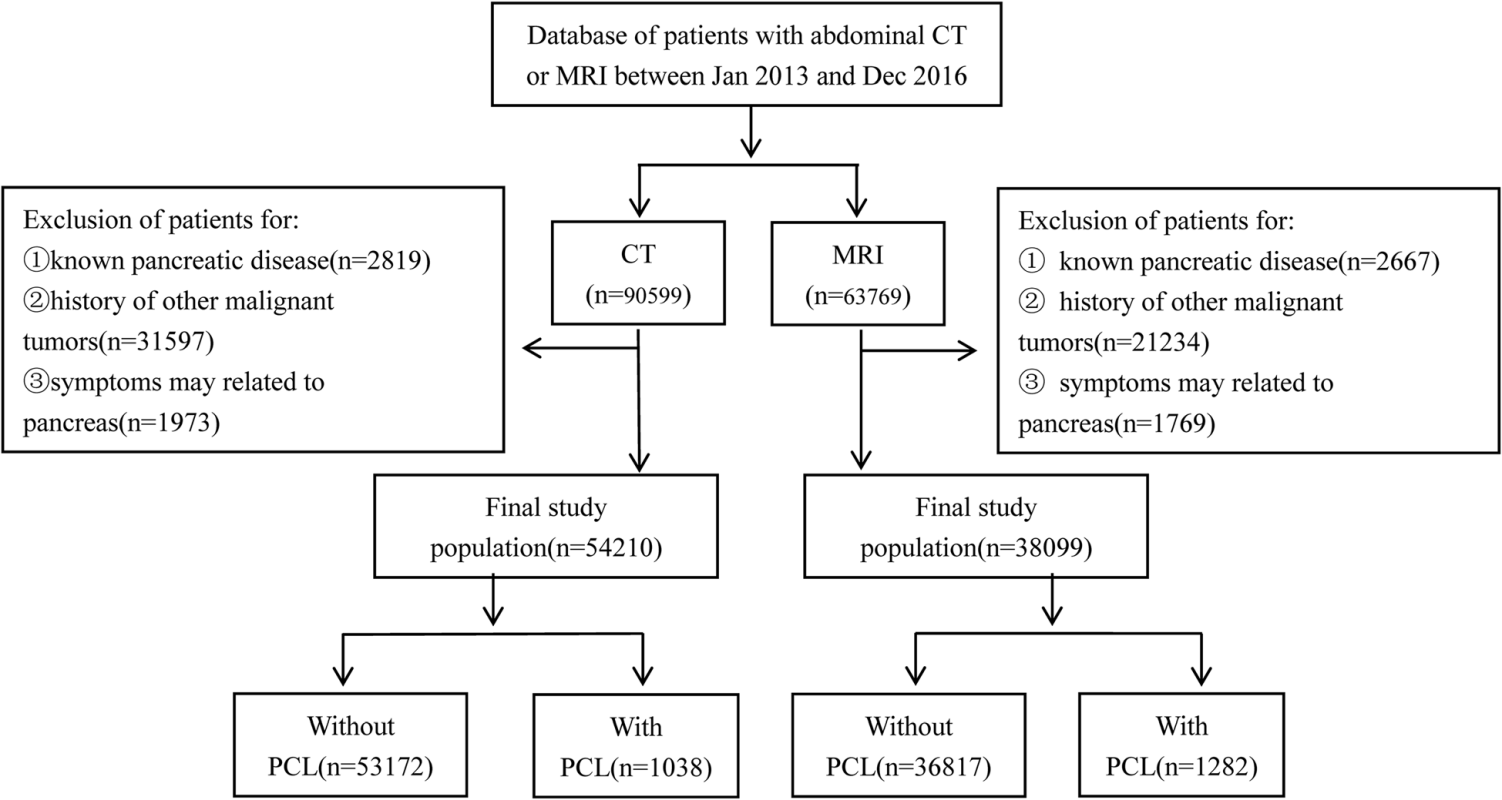
Flow diagram shows inclusion and exclusion criteria for the study

We retrospectively reviewed consecutive patients who underwent an abdominal computed tomography (CT) or magnetic resonance imaging (MRI) examination at our institution during a 4-year period (January1 2013 to December31 2016). Four radiologists with 6 to 7 years of experience in abdominal imaging diagnosis reviewed the diagnostic reports in chronological order by radiology information system (RIS) and picture archiving and communication systems (PACS). If the diagnostic reports included pancreatic cystic lesions, the images would be re-evaluated by two radiologists together. The inclusion criteria for the study included: (a) availability of contrast-enhanced abdominal CT and MRI; (b) diagnosed with pancreatic cysts; (c) enough diagnostic quality of CT or MR images. Exclusion criteria consisted of: (a) patients who had a known or suspected history of pancreatic disease including pancreatic solid tumors, acute/chronic pancreatitis, etc.; (b) patients who had a history of non-pancreatic malignant tumors or cystic pancreatic lesion; (c) CT or MRI examinations for further evaluating PCLs; (d) patients who had non-specific abdominal symptoms which may be related to pancreas. The flowchart for the enrollment of this study is showed in Fig. 1.

### CT and MRI protocol

Contrast-enhanced CT of the pancreas was as a part of the routine abdominal CT. Our study used several multi-slice CT (MSCT) equipment as follows: Siemens Somatom Sensation 16, Somatom Emotion 6, Somatom Definition AS, Siemens Healthcare, Erlangen, Germany; Toshiba Aquilion 64 or Aquilion one 320, Toshiba Medical Systems,Tokyo, Japan; GE Lightspeed VCT 64, GE Healthcare; United imaging Uihct128,China. A gantry rotation time of 0.5 s, a tube current of 150–200 mA, and a peak voltage of 120 kV were used for CT scanners. Contrast-enhanced abdominal CT including pre-contrast, arterial phase (30–35 s), portal venous phase (80s) and delayed phase (3 min) was performed in our hospital. The injection rate of contrast medium (Ultravist 300 mgI/ml or 370 mgI/ml,90-100 ml,Bayer Healthcare, Berlin, Germany) is 3-5 ml/s. Slice thickness was 1-5 mm.

Abdominal MR examinations were performed on either 1.5 T MR scanners (Magnetom Aera or Magnetom Avanto, Siemens Medical Solution, Erlangen, Germany; Uihmr1, United imaging, China) or 3.0 T MR scanners (Magnetom Verio, Siemens Medical Solution, Erlangen, Germany; Signa HDx, GE Healthcare, Milwaukee, USA; Uihmr770, United imaging, China). Avanto and Signa HDx were the most commonly used for abdominal examinations. The conventional abdominal MR included: (1) fat suppressed T2-weighted imaging (Avanto: repetition time (TR)/echo time (TE) = 3500/84 ms; slice thickness = 5 mm; slice gap = 1 mm; field of view (FOV) optimized to patients’ body habitus:285 × 214–308 × 380 mm; matrix = 168 × 320; Signa HDx: TR/TE = 4500–7100/88 ms; slice thickness = 5 mm; slice gap = 2 mm; field of view (FOV) = 400 × 300 mm; matrix = 320 × 224); (2) MR cholangiopancreatography (MRCP) (Avanto: TR/TE = 4500/758 ms; slice thickness = 4 mm; FOV = 340 × 340 mm; matrix = 180 × 320; Signa HDx: TR/TE = 7000/1228 ms; slice thickness = 5 mm; FOV = 300 × 300 mm;matrix = 288 × 288); (3) gradient echo (GRE) T1-weighted in-phase and opposed-phase imaging (Avanto: TR/TE = 6.8/2.35 (in-phase), 4.75 (opposed-phase) msec; slice thickness = 5 mm; slice gap = 1 mm; FOV = 85 × 214–308 × 380 mm; matrix = 180 × 320; Signa HDx: TR/TE = 207/2.31 (in-phase), 3.69 (opposed-phase) msec; slice thickness = 5 mm; slice gap = 1 mm; FOV = 400 × 400 mm; matrix = 192 × 256); (4) dynamic contrast-enhanced imaging (pre-contrast, arterial, portal venous and delayed phases) using 3D-GRE T1 weighted imaging with injection of gadopentetate dimeglumine (Magnevist, Bayer HealthCare, Berlin, Germany) at rate of 2-3 ml/s(Avanto: TR/TE = 5.04/2.31 msec, slice thickness = 3 mm, no slice gap, matrix = 250 × 512; Signa HDx: TR/TE = 4.1/1.4 msec, slice thickness = 3 mm, no slice gap, matrix = 200 × 352).

### Data collection

**Fig. 2:**
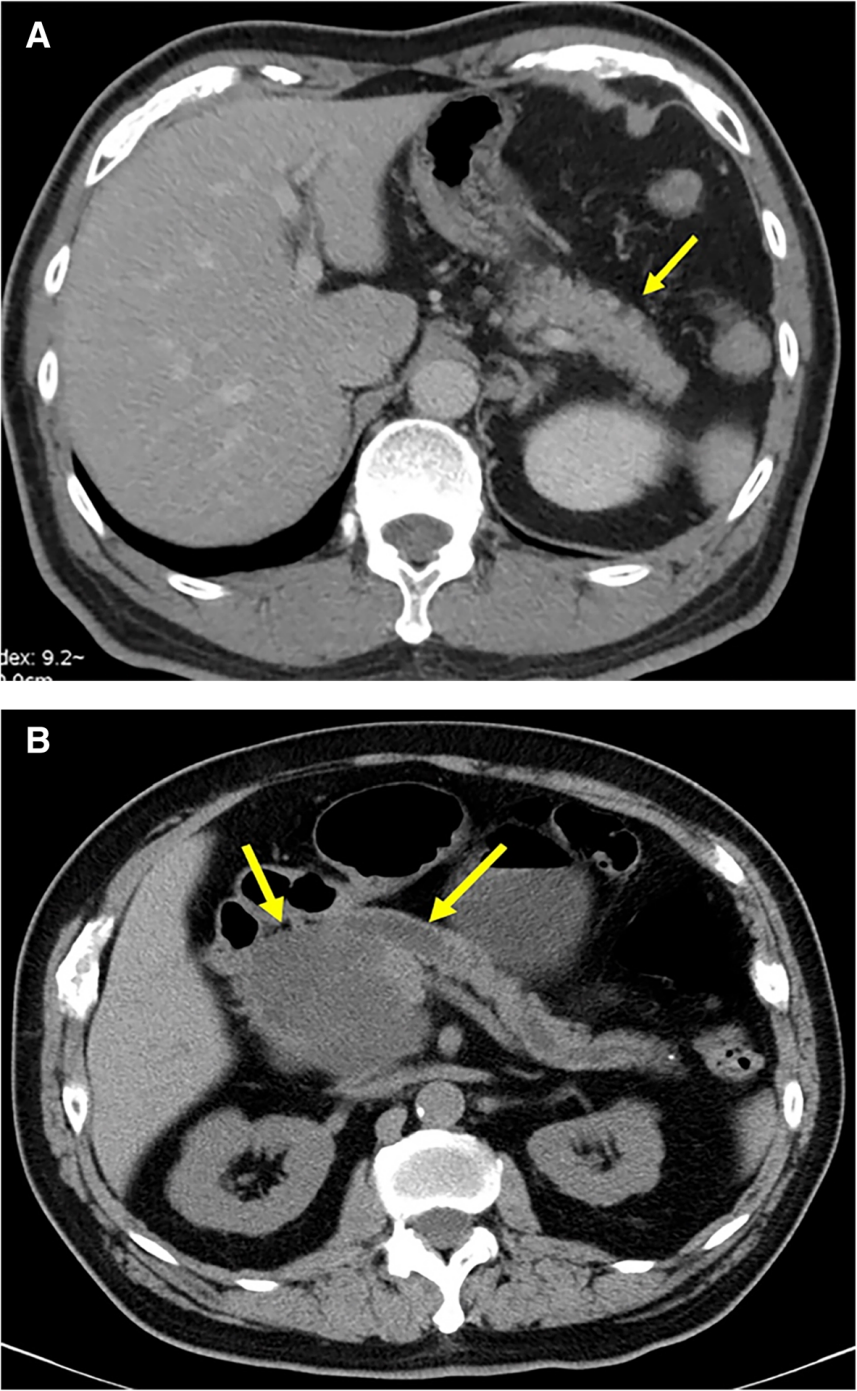
Imaging features of PCLs detected by CT. **a** a patient with a small (6 mm) cyst in the body of pancreas. Transverse image from contrast-enhanced CT scan shows slightly low-density lesion (arrow) in the body of the pancreas and is easy to be misdiagnosed as pancreatic fat infiltration. **b** a patient with a cyst in the head of pancreas. Transverse image shows a low-density lesion in the head of pancreas with dilation of main pancreatic duct (arrow)

**Fig. 3:**
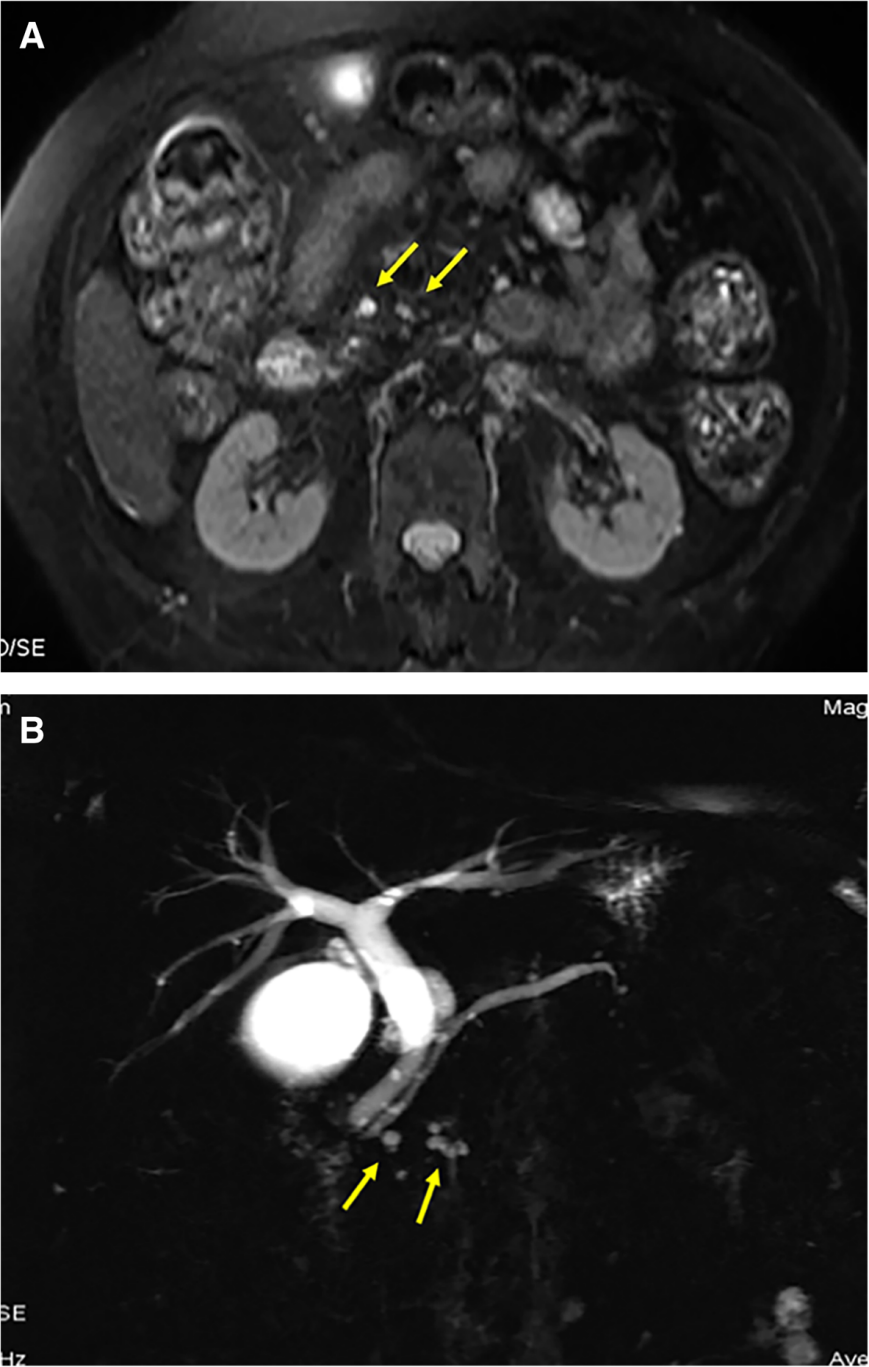
Imaging features of PCLs detected by MRI. A patient with multiple small cysts in the head of pancreas. **a**, and **b**, transverse T2-weighted fat-suppressed turbo-spin-echo (TSE) MR images and MR cholangiopancreatography show multiple cysts (arrow) with well-defined homogeneous signal intensity in the head of pancreas

The following data were collected for each patient: age, sex and features of PCLs (size, location, number of cysts, communication to pancreatic duct and worrisome features such as enhancing mural nodule < 5 mm, thickened enhanced cyst walls, MPD size≥5 mm, abrupt change in the MPD caliber with distal pancreatic atrophy etc. [10]). If the lesions were multiple, only the largest one was recorded. According to guidelines, if patient has the absolute or relative indications for surgery such as jaundice, positive cytology, pancreatic duct ≥10 mm and enhancing mural nodules ≥5 mm etc., he/she should undergo surgery and/or be referred to a multidisciplinary group for further evaluation [10–12]. If not, they could be followed up. If patient had undergone surgical resection and the pathologic result would be recorded. Samples of the PCLs detected by CT and MRI we reviewed are presented in Figs. 2 and 3.

### Statistical analysis

Quantitative data are presented by median values with range or mean of standard deviations according to distributional properties. The overall and annual detection rate of pancreatic cysts for CT and MRI were calculated respectively. Furthermore, annual detection rates between CT and MRI would be compared. Then, we evaluated and compared the rates of surgery for pancreatic cysts in CT and MRI. The subjects were stratified by age (≤29,30-39,40-49,50-59,60-69,70–79,≥80), gender and location. The chi-square was used for categorical variable for comparing CT with MRI and calculating changes of detection rate, while independent t test was applied for continuous variables. A *P* value < 0.05 was considered statistically significant. All statistical analyses were performed using SPSS software (version 24.0; SPSS, Chicago, IL).

## Results

### Patient characteristics

A total of 90,599 abdominal CT and 63,769 MRI scans were performed between January 2013 and December 2016. Among of them, 54,210 of CT and 38,099 of MRI scans met our selection criteria. The mean age of the 54,210 individuals for CT and the 38,099 for MRI at that time was 55.16 ± 14.64 years and 53.94 ± 14.14 years respectively, and 50% were female in both CT and MR scans. Overall incidental detection rate was 2.51% (2320/92309) for PCLs on CT and MRI, with 10.2% (237/2320) patients with PCLs having multiple lesions. Furthermore, the PCLs were identified in 1038 patients with CT and 1282 patients with MRI examinations, representing the prevalence was 1.91% (95% CI, 1.8–2.0%) for CT and 3.36% (95% CI, 3.2–3.5%) for MRI, respectively.

Of all individuals with PCLs detected by CT, the median age of patients was 62 years (range, 8-96y) in comparison with 63 years (range, 16-94y) in the group with MRI, and about 60–61% of whom were women for CT and MRI. The mean age (62.26 ± 14.14y of CT and 60.74 ± 13.86y of MRI) of populations with PCLs was older than the mean age (55.16 ± 14.64 of CT and 53.94 ± 14.14 of MRI) of those without PCLs (*P* < .001, respectively). The prevalence of PCLs which were found by CT was 1.54% (418/27143) in males, compared with 2.30% (620/27067) in females with significant difference (*P* < 0.001), as well as MRI (2.63% = 498/18954 in males, 4.10% = 784/19145 in females, *P* < 0.001). Patient baseline characteristics were summarized in the Table 1.

**Table 1:** Patient baseline characteristics and detection rate by age and gender

	CT	MRI
Age (y)	62.26 ± 14.14	60.74 ± 13.86
Gender (M/F)	418/620	498/784
Location of PCLs		
Uncinate	12.0%(125/1038)	10.6%(136/1282)
Head	20.2%(210/1038)	22.8%(292/1282)
Neck	13.3%(138/1038)	13.6%(175/1282)
Body	32.1%(333/1038)	31.9%(409/1282)
Tail	22.4%(232/1038)	21.1%(270/1282)
Detection rate by Gender		
Male	1.54% (418/27143)	2.63%(498/18954)
Female	2.30% (620/27067)	4.10%(784/19145)
Detection rate by Age		
≤ 29	0.40%(24/6000)	0.58%(36/6196)
30–39	0.83%(47/5657)	1.56%(65/4176)
40–49	1.40%(106/7570)	2.83%(139/4917)
50–59	1.80%(225/12500)	3.50%(292/8349)
60–69	2.21%(314/14236)	4.49%(422/9391)
70–79	3.46%(208/6007)	6.06%(232/3827)
≥ 80	5.09%(114/2240)	7.72%(96/1243)

Abbreviations: *CT* computed tomography, *MRI* magnetic resonance imaging, *M* male, *F* female, *PCLs* pancreatic cystic lesions

### Cyst characteristic

**Fig. 4:**
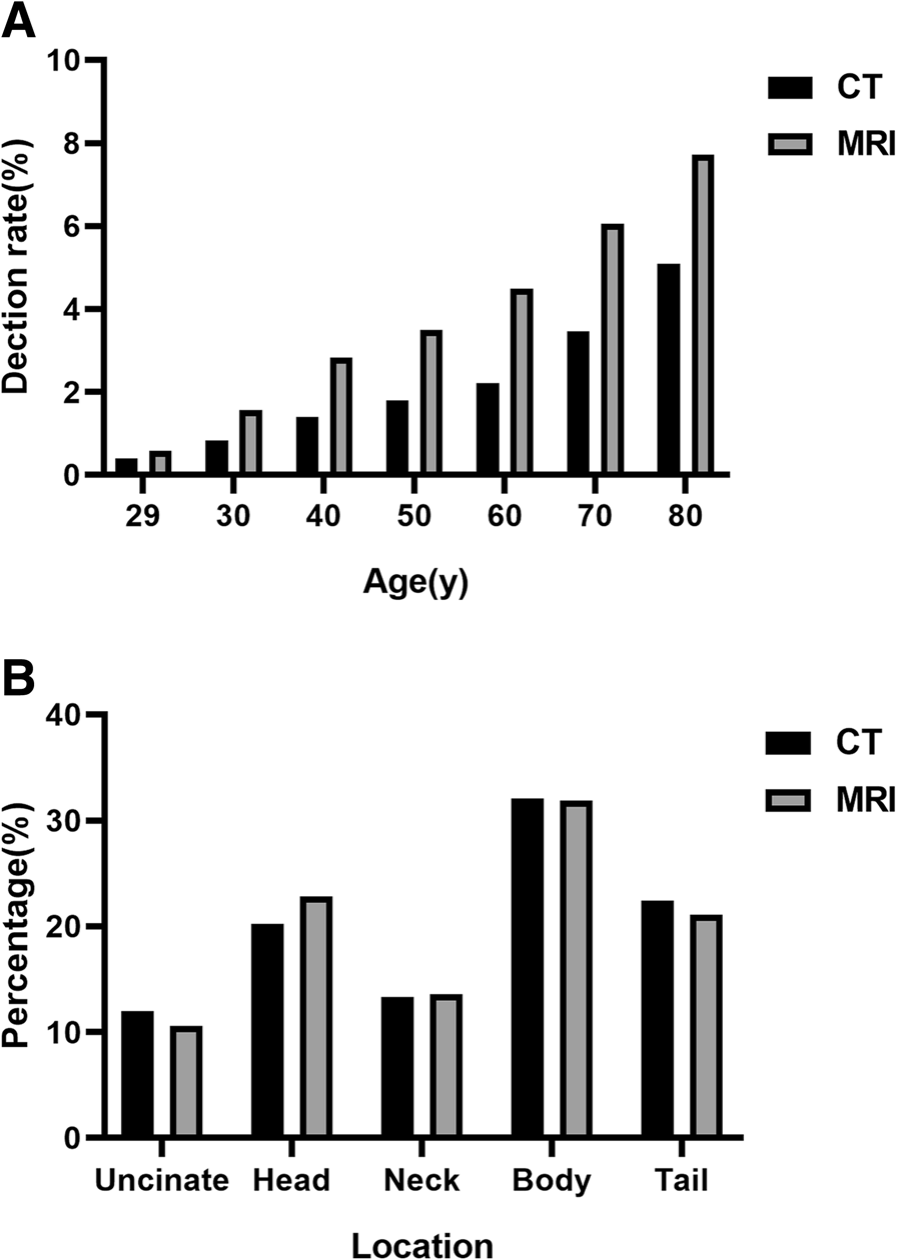
Detection rate by age and locations of PCLs between CT and MRI. **a** Figure shows the detection rate of PCLs increased with increasing age. **b** Figure shows the locations of PCLs detected by CT and MRI

The prevalence of PCLs increased with increasing age (*P* < 0.001). The maximal diameter of the largest lesions in CT and MRI varied from 1 mm to 165 mm (median size, 12 mm and 13 mm, respectively). PCLs (or the location of the largest one if lesions were multiple) detected by CT were located throughout the pancreas: uncinate (12.0%, 125/1038), head (20.2%, 210/1038), neck (13.3%, 138/1038), body (32.1%, 333/1038), tail (22.4%, 232/1038). The respective numbers for PCLs detected by MRI were 10.6%(136/1282), 22.8%(292/1282), 13.6%(175/1282), 31.9%(409/1282), 21.1%(270/1282). Furthermore, female patients had an increased frequency of PCLs within the distal gland (body and tail, *P* < 0.001 with CT and MRI, respectively). The location of PCLs and detection rate by age and gender were also provided in the Table 1 and Fig. 4.

The dilatation of the pancreatic duct was reported about 11% in patients both for CT and MRI. The portion of lesions communicated with the pancreatic duct in MRI was 16.5% (211/1282), compared to 10.6% (110/1038) in CT (*P* < 0.001). The detection rate of multiple lesions in CT and MRI was 8.0 and 12.0% (*P* = 0.001), respectively. About 1.4 and 3.1% of cysts had solid portion such as the thickened septa or mural nodularity which may represent malignant degeneration in CT and MRI (*P* = 0.008), respectively. The detection rate of worrisome features in CT and MRI was 25.7 and 31.7% (*P* = 0.001), respectively. Characteristics of pancreatic cysts were summarized in the Table 2.

**Table 2:** Characteristics of pancreatic cystic lesions detected by CT and MRI from 2013 to 2016

Characteristics	CT	MRI	*P* value
Mean size (mm)	18.99 ± 17.45	17.45 ± 15.74	0.028
Single/Multiple	955/83	1128/154	0.001
Dilatation of PD	11.4% (118/1038)	10.8% (138/1282)	0.645
Communication to PD	10.6% (110/1038)	16.5% (211/1282)	< 0.001
Multilocular	7.5% (78/1038)	8.6% (110/1282)	0.350
Solid portion	1.4% (15/1038)	3.1% (40/1282)	0.008

Abbreviations: *CT* computed tomography, *MRI* magnetic resonance imaging, *PD* pancreatic duct

### CT versus MRI

**Fig. 5:**
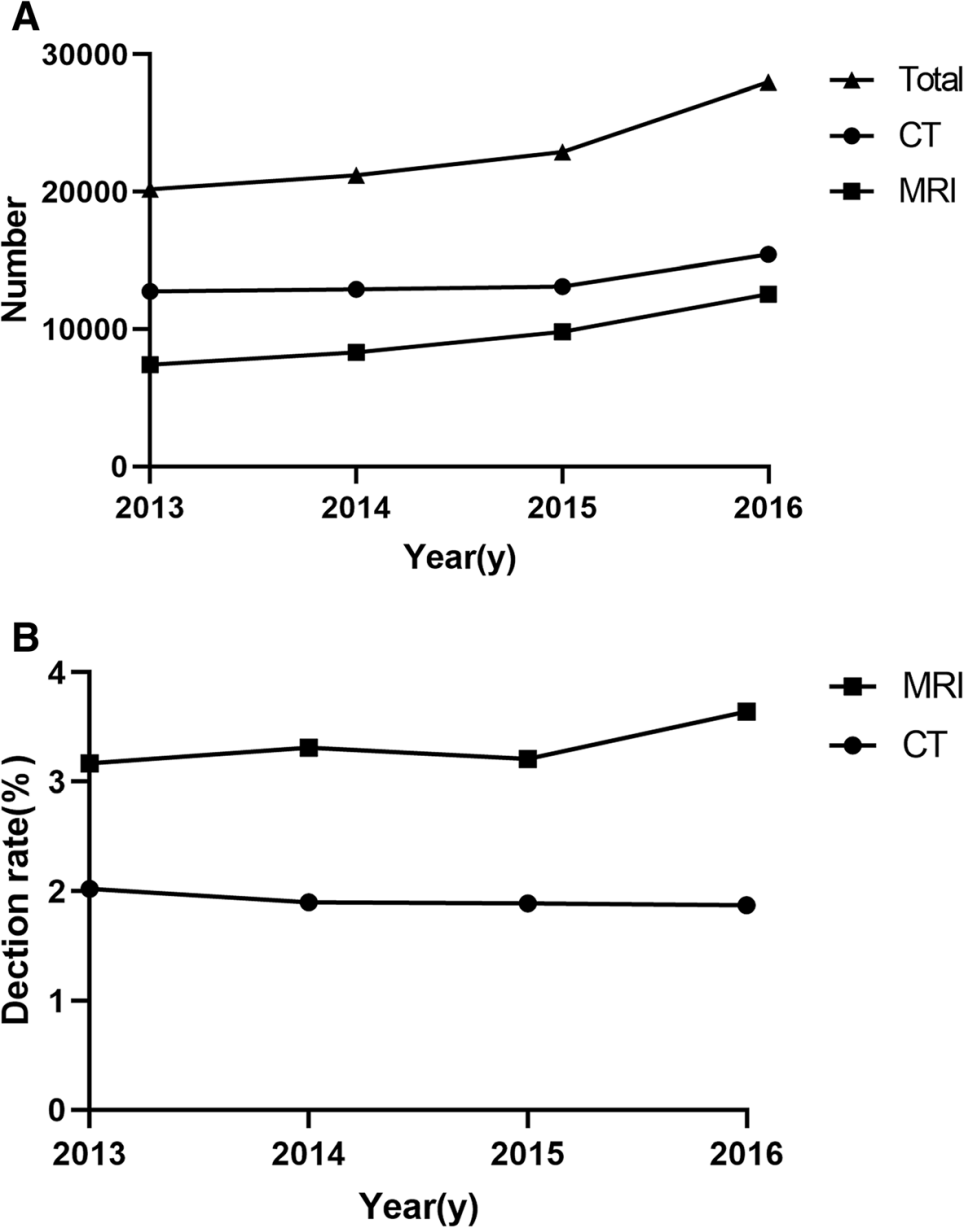
Numbers of CT and MRI examinations and detection rate of PCLs from 2013 to 2016. **a** Figure shows the annual number of CT and MRI examinations between 2013 and 2016. The number was increasing every year. **b** Figure shows the detection rate of PCLs on CT and MRI from 2013 to 2016. There was no significant difference in the annual detection rate of PCLs in CT or MRI. The detection rate of MRI was higher than CT

Although population who underwent CT and MRI from 2013 to 2016 was increasing, there was no significant difference in the prevalence of pancreatic cystic lesions in the CT group (*P* = 0.796). The difference in MRI was also not obvious from 2013 to 2016 (*P* = 0.213). After 2013, the prevalence of pancreatic cysts between CT and MRI had a statistically significant difference (*P* < 0.001). Moreover, the prevalence of PCLs smaller than 20 mm in MRI was higher than CT (2.56% vs.1.20%, *P* < 0.001), but there was no difference in the prevalence of PCLs larger than or equal to 20 mm between CT and MRI (0.71% vs.0.80%, *P* = 0.122). The difference between CT and MRI from 2013 to 2016 was shown in the Table 3 and tendency of the detection rate was provided in the Fig. 5.

**Table 3:** Detecting rate of pancreatic cysts of CT and MRI from 2013 to 2016

	2013		*P* value	2014		*P* value	2015		*P* value	2016		*P* value
	CT	MRI		CT	MRI		CT	MRI		CT	MRI	
Age (y)	63.21 ± 14.25	61.06 ± 14.86	...	62.78 ± 13.65	61.37 ± 13.44	...	62.43 ± 14.12	60.81 ± 13.51	...	60.83 ± 14.43	60.15 ± 13.83	...
Gender (M/F)	93/165	81/154	...	104/141	122/153	...	106/141	129/186	...	115/173	166/291	...
Number (%)	258/12774 (2.02%)	235/7413 (3.17%)	< 0.001	245/12901 (1.90%)	275/8319 (3.31%)	< 0.001	247/13103 (1.89%)	315/9800 (3.21%)	< 0.001	288/15432 (1.87%)	457/12567 (3.64%)	< 0.001
Median Size (mm)	12.0	12.0	...	14.0	13.0	…	15.0	14.0	...	12.0	11.0	...
< 20 mm	165	170	< 0.001	151	209	< 0.001	150	236	< 0.001	185	361	< 0.001
≥20 mm	93	65	0.247	94	66	0.595	97	79	0.572	103	96	0.339

Abbreviations: *CT* computed tomography, *MRI* magnetic resonance imaging, *M* male, *F* female

### Treatment characteristics

As before, if patient has the absolute or relative indications for surgery, he/she should undergo surgery or be referred to the multidisciplinary group for further evaluation [10–12]. Finally, 312 cases underwent surgical resection. The maximum diameter of all resected lesions was larger than or equal to 20 mm and there was no difference in mean size between CT and MRI (*P* = 0.857). Interestingly, although there was no significant difference in the prevalence of cysts larger than or equal to 20 mm between CT and MRI (0.71% vs.0.80%, *P* = 0.122), we found significant difference for surgical resection rates of PCLs between CT and MRI (37.0% 143/387 vs. 55.2% 169/306, *P* < 0.001). The pathologic results were: benign (*n* = 283, 90.7%), borderline (*n* = 2, 0.6%), malignant (*n* = 27, 8.7%). The incidence of malignancy in the CT group was 4.9%, compared to 13.0% in MRI (*P* = 0.014).

## Discussion

The present study demonstrated that: (a) the detection rate of incidental PCLs in large-scale general population increased with increasing age and showed a significant difference between CT (1.91%) and MRI (3.36%). MRI detected more small PCLs (< 20 mm) in comparison with CT and female had a slightly more frequency of cyst than the male. (b) the detection rate of PCLs was stabilized with increasing volumes of CT and MRI examinations from 2013 to 2016. (c) patients with PCLs(≥20 mm) detected by MRI had a higher rate of surgical resection compared to CT group (55.2% vs. 37.0%) while there was no significant difference for the detection rate of PCL (≥20 mm) between MRI and CT.

The detection rate of PCLs in our study (1.91% of CT and 3.36% of MRI) was in keeping with previous large-scale study from Chang et al. [13], who reported the crude prevalence rate was 2.1% among asymptomatic healthy population (21,745 individuals) by using CT. In addition, the detection rate of PCLs in our study increased with age like the previous studies [1–5]. Only 0.78%(172/22029) was identified in individuals younger than 40 years. Whereas 4.88%(650/13317) of those older than or equal to 70 years had PCLs.

We found that female patients had a slightly more frequency of cysts than males. As we all know, the mucinous cystic neoplasms (MCNs) which is one of pancreatic cystic lesions are more commonly in female because MCNs were originally defined as tumors with mucin-producing columnar epithelium supported by ovarian stroma [14]. Another theory reported by Zamboni et al. was that the stimulation of endodermal immature stroma by female hormones or that primary yolk cells are implanted in the pancreas, as buds of the genital tract and dorsal pancreas are adjacent to each other during embryogenesis [15].

One of interests is that the detection rate of PCLs did not change more despite CT or MR scan volumes increasing over time. This was mainly due to large-scale population in our study and the incidence of PCLs was steady over time. As the number of individuals reaches a certain substantial amount, the detection rate is close to the real incidence.

MRI was more sensitive for PCLs than CT (3.36% vs.1.91%). The higher prevalence on MRI was primarily due to increased detection of small cystic lesions smaller than 20 mm. Another noteworthy finding was that although there was no significant difference in prevalence of PCLs larger than or equal to 20 mm between MRI and CT, the rate of surgical resection of PCLs in MRI group was higher than CT (55.2% vs. 37.0%). The disparity between CT and MRI may be ascribed to the advantage of soft-tissue contrast and the use of MRCP [16, 17], which could better show the internal morphology of the PCLs such as the thickened septa or mural nodularity. This was line with the rate of malignancy confirmed by postoperative pathology. Although the natural history of pancreatic cystic lesions was not clear, some previous studies had suggested that small (≤30 mm), incidental, simple cysts were less likely to be malignant and could choose to follow-up [18–20]. The recent guideline noted that if the cyst was small (< 20 mm), asymptomatic, simple, surveillance by MRI was recommended [21], due to non-invasiveness, lack of radiation and greater accuracy in identifying communication with pancreatic duct for MRI or MRCP, which was consistent with our study that MRI detected more PCLs smaller than 20 mm compared with CT.

The limitation of this study was that we studied on the prevalence by reviewing the imaging reports of CT and MRI rather than directly evaluating all of the images because it was highly time-consuming for reviewing 90,599 CT and 63,769 MRI scans. Overlooking the small and simple cysts or originally unreported may result in underestimating the actual prevalence. de Jong et al. [1] believed that re-evaluation of MR images did not a reveal a significant difference from original MR reports with similar prevalence of PCLs (2.4%) in a large-scale population as our study (3.36%). In addition, we obtained the patients from a single tertiary care university hospital. Therefore, selection bias in this retrospective study cannot be avoided. In our study, we calculated the prevalence of PCLs with strict inclusion criteria, but more importantly, we compared the differences between CT and MRI in the population from the same hospital which may limit this influence. Moreover, when the cysts were multiple, the largest one would be selected as a model for analysis. Because the largest lesion is more characteristic and will influence the clinical decision-making.

## Conclusions

The detection rate of PCLs on CT and MRI (1.91 and 3.36%, respectively) was steady despite increasing scan volumes over time. Female had slightly more frequency of cyst than male. MRI detected more PCLs smaller than 20 mm compared with CT. For lesions larger than or equal to 20 mm, MRI could display a greater level of internal details than CT, which could help clinicians to make management decisions.

## Abbreviations

CT: Computed tomography
MRCP: Magnetic resonance cholangiopancreatography
MRI: Magnetic resonance imaging
MSCT: Multi-slice computed tomography
PACS: Picture archiving and communication systems
PCLs: Pancreatic cystic lesions
PD: Pancreatic duct
RIS: Radiology information system
TSE: Turbo-spin-echo

## References

[1] de Jong K, Nio CY, Hermans JJ, Dijkgraaf MG, Gouma DJ, van Eijck CH, van Heel E, Klass G, Fockens P, Bruno MJ (2010). High prevalence of pancreatic cysts detected by screening magnetic resonance imaging examinations. Clin Gastroenterol Hepatol.

[2] Lee KS, Sekhar A, Rofsky NM, Pedrosa I (2010). Prevalence of incidental pancreatic cysts in the adult population on MR imaging. Am J Gastroenterol.

[3] Laffan TA, Horton KM, Klein AP, Berlanstein B, Siegelman SS, Kawamoto S, Johnson PT, Fishman EK, Hruban RH (2008). Prevalence of unsuspected pancreatic cysts on MDCT. AJR Am J Roentgenol.

[4] Zhang XM, Mitchell DG, Dohke M, Holland GA, Parker L (2002). Pancreatic cysts: depiction on single-shot fast spin-echo MR images. Radiology.

[5] Martinez B, Martinez JF, Aparicio JR (2018). Prevalence of incidental pancreatic cyst on upper endoscopic ultrasound. Ann Gastroenterol.

[6] Moris M, Bridges MD, Pooley RA, Raimondo M, Woodward TA, Stauffer JA, Asbun HJ, Wallace MB (2016). Association between advances in high-resolution cross-section imaging technologies and increase in prevalence of pancreatic cysts from 2005 to 2014. Clin Gastroenterol Hepatol.

[7] Kromrey ML, Bulow R, Hubner J, Paperlein C, Lerch MM, Ittermann T, Volzke H, Mayerle J, Kuhn JP (2018). Prospective study on the incidence, prevalence and 5-year pancreatic-related mortality of pancreatic cysts in a population-based study. Gut.

[8] Gardner TB, Glass LM, Smith KD, Ripple GH, Barth RJ, Klibansky DA, Colacchio TA, Tsapakos MJ, Suriawinata AA, Tsongalis GJ (2013). Pancreatic cyst prevalence and the risk of mucin-producing adenocarcinoma in US adults. Am J Gastroenterol.

[9] Scheiman JM, Hwang JH, Moayyedi P (2015). American gastroenterological association technical review on the diagnosis and management of asymptomatic neoplastic pancreatic cysts. Gastroenterology.

[10] Tanaka M, Fernandez-Del Castillo C, Kamisawa T, Jang JY, Levy P, Ohtsuka T, Salvia R, Shimizu Y, Tada M, Wolfgang CL (2017). Revisions of international consensus Fukuoka guidelines for the management of IPMN of the pancreas. Pancreatology.

[11] Vege SS, Ziring B, Jain R, Moayyedi P (2015). Clinical guidelines C, American gastroenterology a: American gastroenterological association institute guideline on the diagnosis and management of asymptomatic neoplastic pancreatic cysts. Gastroenterology.

[12] European Study Group on Cystic Tumours of the P (2018). European evidence-based guidelines on pancreatic cystic neoplasms. Gut.

[13] Chang YR, Park JK, Jang JY, Kwon W, Yoon JH, Kim SW (2016). Incidental pancreatic cystic neoplasms in an asymptomatic healthy population of 21,745 individuals: large-scale, single-center cohort study. Medicine (Baltimore).

[14] Compagno J, Oertel JE (1978). Mucinous cystic neoplasms of the pancreas with overt and latent malignancy (cystadenocarcinoma and cystadenoma). A clinicopathologic study of 41 cases. Am J Clin Pathol.

[15] Zamboni G, Scarpa A, Bogina G, Iacono C, Bassi C, Talamini G, Sessa F, Capella C, Solcia E, Rickaert F (1999). Mucinous cystic tumors of the pancreas: clinicopathological features, prognosis, and relationship to other mucinous cystic tumors. Am J Surg Pathol.

[16] Song SJ, Lee JM, Kim YJ, Kim SH, Lee JY, Han JK, Choi BI (2007). Differentiation of intraductal papillary mucinous neoplasms from other pancreatic cystic masses: comparison of multirow-detector CT and MR imaging using ROC analysis. J Magn Reson Imaging.

[17] Waters JA, Schmidt CM, Pinchot JW, White PB, Cummings OW, Pitt HA, Sandrasegaran K, Akisik F, Howard TJ, Nakeeb A (2008). CT vs MRCP: optimal classification of IPMN type and extent. J Gastrointest Surg.

[18] Sahani DV, Saokar A, Hahn PF, Brugge WR, Fernandez-Del Castillo C (2006). Pancreatic cysts 3 cm or smaller: how aggressive should treatment be?. Radiology.

[19] Handrich SJ, Hough DM, Fletcher JG, Sarr MG (2005). The natural history of the incidentally discovered small simple pancreatic cyst: long-term follow-up and clinical implications. AJR Am J Roentgenol.

[20] Megibow AJ, Lombardo FP, Guarise A, Carbognin G, Scholes J, Rofsky NM, Macari M, Balthazar EJ, Procacci C (2001). Cystic pancreatic masses: cross-sectional imaging observations and serial follow-up. Abdom Imaging.

[21] Elta GH, Enestvedt BK, Sauer BG, Lennon AM (2018). ACG clinical guideline: diagnosis and Management of Pancreatic Cysts. Am J Gastroenterol.

